# Effects of Remimazolam Anesthesia with Two Induction Doses on Hemodynamics and Recovery Profile in Older Patients: Comparison with Propofol Anesthesia

**DOI:** 10.3390/jcm12165285

**Published:** 2023-08-14

**Authors:** Tae Kwang Kim, Hyun Jeong Kwak, Wol Seon Jung, Gyu Bin Choi, Sung Yong Park, Jong Yeop Kim

**Affiliations:** 1 Department of Anesthesiology and Pain Medicine, Ajou University School of Medicine, Suwon 16499, Republic of Korea; 2 Department of Anaesthesiology and Pain Medicine, Gachon University Gil Medical Center, Incheon 21565, Republic of Korea

**Keywords:** general anesthesia, elderly, remimazolam, propofol, hemodynamics

## Abstract

Remimazolam has advantages such as hemodynamic stability and rapid onset. We investigated the effects of induction doses on hemodynamics and recovery profiles for remimazolam compared to propofol in older patients. Sixty-nine patients aged >65 years were randomly assigned to either the propofol anesthesia group (P group) or the remimazolam anesthesia group with an induction dose of 6 mg/kg/h (R6 group) or 12 mg/kg/h (R12 group), followed by 1 mg/kg/h. P group was anesthetized with 4 µg/mL of propofol effect-site concentration (Ce) with target-control infusion, followed by 2.5–3 µg/mL of Ce. The primary outcome was the difference between the baseline mean arterial pressure (MAP) and the lowest MAP during anesthesia (ΔMAP). ΔMAP was comparable between the P, R6, and R12 groups (43.8 ± 13.8 mmHg, 39.2 ± 14.3 mmHg, and 39.2 ± 13.5 mmHg, *p* = 0.443). However, the frequencies of vasoactive drug use were 54.5%, 17.4%, and 30.4% (*p* = 0.029), and the median doses of ephedrine 3 (0–6) mg, 0 (0–0) mg, and 0 (0–0) mg (*p* = 0.034), which were significantly different. This study showed remimazolam anesthesia with an induction dose of 6 mg/kg/h, rather than 12 mg/kg/h, could reduce the requirement for vasoactive drugs compared to propofol anesthesia.

## 1. Introduction

Intraoperative hypotension frequently manifests during surgery. This can lead to organ ischemia and oxidative stress, heightening the risk of subsequent organ dysfunction and amplifying postoperative complications. Intraoperative mean arterial pressure (MAP) below 65 mmHg correlates with both myocardial and renal injuries, with the associated risks escalating in tandem with prolonged hypotensive episodes [1,2,3]. Propofol is the most frequently used intravenous anesthetic agent and can cause injection pain and hemodynamic instability [4]. Propofol induces dose-dependent hypotension by reducing systemic vascular resistance and has negative inotropic effects on the myocardium [5]. Cardiovascular suppression by propofol is more sensitive in older patients; therefore, it should be used at a reduced dose [6]. Remimazolam is a recently developed benzodiazepine characterized by a quick onset of action and undergoes rapid metabolism by a nonspecific esterase to form an inactive metabolite [7,8]. A recent randomized study showed that remimazolam anesthesia at two induction doses (6 and 12 mg/kg/h) had efficacy and safety comparable to those of propofol anesthesia at an induction dose of 2.0–2.5 mg/kg [9]. Moreover, remimazolam has a lower incidence of intraoperative hypotension than propofol [10]. Regarding recovery profiles, previous studies have shown inconsistent results on the emergence time and quality of remimazolam and propofol anesthesia and sedation [9,11,12]. Few studies have compared the hemodynamic and recovery profiles of remimazolam and propofol anesthesia based on induction dose of remimazolam in elderly patients [11,12,13,14,15,16]. Therefore, in this study, we compared the intraoperative hemodynamic changes and recovery indices, such as time to emergence and time to extubation, when propofol and remimazolam were used in combination with remifentanil in older patients.

## 2. Materials and Methods

**Figure 1:**
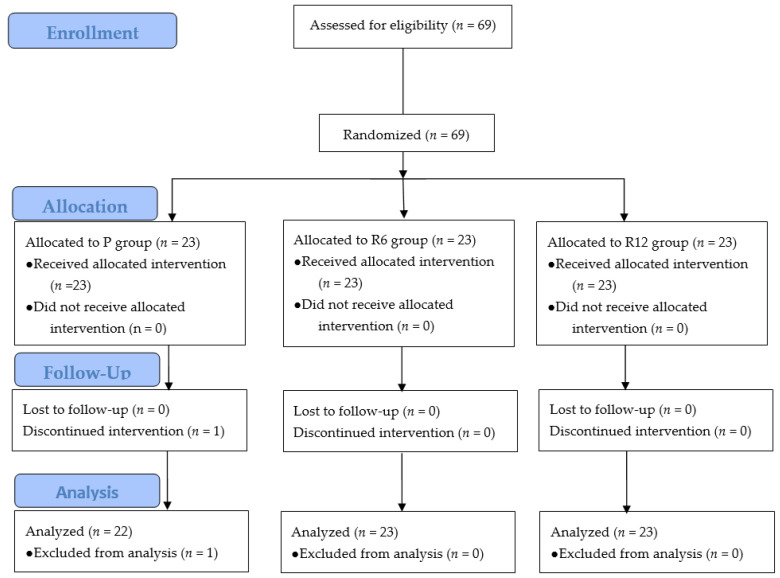
Consort flow diagram of this study.

After receiving approval from the institutional review board (AJIRB-MED-INT-21-651), this study was registered on clinicaltrials.gov (NCT05201300). This study was conducted at a tertiary hospital between March 2022 and December 2022. All participants provided written informed consent prior to participation. The study was conducted in accordance with the 2013 Declaration of Helsinki. This study included patients aged > 65 years with an American Society of Anesthesiologists (ASA) physical status of 1 or 2. All participants underwent surgical procedures that required general anesthesia at a tertiary hospital. Hemodynamically stable elective surgeries were included. The exclusion criteria included preoperative uncontrolled hypertension, severe cardiovascular disease, chronic obstructive pulmonary disease, asthma, severe liver dysfunction, kidney dysfunction, and neurological disorders. Emergency surgery, heart surgery, and trauma surgery that could be hemodynamically unstable were also excluded. A total of 69 patients were enrolled in the study. One patient in the P group was excluded because of unexpected hemodynamic instability during surgery. Therefore, we analyzed data from 68 patients (Figure 1). There were no significant differences between the groups regarding sex, age, height, and weight of the patients (Table 1). The study participants were divided into three groups through a randomization process derived from www.ramdom.org (accessed on 2 March 2022): propofol anesthesia group (P group, *n* = 23), remimazolam anesthesia with an induction dose of 6 mg/kg/h (R6 group, *n* = 23), and remimazolam anesthesia with an induction dose of 12 mg/kg/h (R12 group, *n* = 23). The patients were not administered any premedication. Intraoperative monitoring devices were used, including electrocardiography (ECG), non-invasive blood pressure, and pulse oximetry devices. We used bispectral index (BIS) monitoring (Covidien LLC, Mansfield, MA, USA) and Tetragraph^®^ for neuromuscular monitoring (Senzime, Uppsala, Sweden). Patients in the P group received propofol anesthesia via a target-controlled infusion (TCI) device, Orchestra^®^ (Fresenius-Vial, Brezins, France) with an induction dose of an effect-site concentration (Ce) of 4 µg/mL. Patients in groups R6 and R12 were anesthetized with continuous intravenous infusion for 2 min or until loss of consciousness at induction doses of 6 and 12 mg/kg/h, respectively. All anesthesia inductions were combined with 4 ng/mL of remifentanil Ce, followed by 2.5–3 ng/mL of remifentanil Ce. Propofol and remifentanil were administered with the Marsh and Minto models. The study defined “Loss of consciousness (LoC)” as the point at which the patient no longer responded to verbal commands to open their eyes. After the patients had lost consciousness, they were administered a dose of 0.6 mg/kg of rocuronium, followed by bag-mask ventilation (BMV) using 100% oxygen. After 2 min of BMV and confirming zero train-of-four (TOF) and a BIS below 60, endotracheal intubation was performed with a 7.5 mm endotracheal tube (ETT) for man and a 7.0 mm ETT for woman. The cuff pressure of ETT was maintained at 25 mmHg using an ETT cuff pressure manometer, Ho-Lo hand pressure gauge. (VBM Medizintechnik GmbH, Munich, Germany). For the maintenance of anesthesia, propofol was adjusted to 2.5–3 µg/mL of Ce, and remimazolam was adjusted to 0.8–1.2 mg/kg/h. This study aimed to maintain the BIS value within the range of 40–60. Mechanical ventilation was regulated by maintaining end-tidal CO_2_ (EtCO_2_) levels within the range of 35–40 mm Hg. Hypotension (mean arterial pressure [MAP] under 55 mmHg twice a row) was treated with 8 mg of ephedrine or 60 µg of phenylephrine. Bradycardia (45 beats/min, lasting > 1 min) was treated with 0.2 mg of glycopyrrolate or 0.5 mg of atropine. When the surgery ended, drug infusion was stopped, and if a T2 twitch of TOF was observed, sugammadex 2 mg/kg was administered for neuromuscular block reversal. Mechanical ventilation was stopped, and BMV was performed with 100% oxygen to maintain 40–45 mmHg of EtCO_2_. Extubation was performed when the TOF ratio was over 90%. We also checked whether patients could open their eyes spontaneously or by verbal order, lift their heads, and breathe with an appropriate tidal volume and respiratory frequency. After extubation, 100% oxygen was administered using a facial mask. We checked the time to eye-opening, time to extubation, and the incidence and grade of emergence cough during the extubation period. The cough severity scale used in this study rated coughing as 0 for no cough, 1 for a single cough, 2 for multiple non-sustained coughing episodes, and 3 for sustained and repetitive coughing with a head lift. The primary outcome was the difference between the baseline MAP and the lowest MAP during anesthesia (ΔMAP). Secondary outcomes included the administration of vasoactive medications during anesthesia, the dosages of vasopressors utilized, the duration from the end of the drug infusion to the moment the patient opened their eyes, and the time until extubation was completed. Hemodynamic parameters, including mean blood pressure and heart rate, were measured at baseline (T1), 1 min after anesthetic induction (T2), immediately before intubation (T3), 1 min after intubation (T4), 5 min intervals during anesthesia (T5–T8), at the end of the operation (T9), 1 min after extubation (T10), and at the end of anesthesia (T11).

### Statistical Analysis

According to the findings of a prior investigation conducted by Hino et al. [17], ΔMAP during anesthesia using remimazolam or propofol in middle-aged and older participants was 45 ± 19 (mean ± standard deviation [SD]) mmHg. Assuming that a difference of 20 mmHg or more is clinically significant during anesthesia using remimazolam [17], the number of patients required for each of the three groups after one-way analysis of variance (ANOVA) with Bonferroni correction was 21 per group. Assuming a dropout rate of 10%, 69 patients were included in the study (*n* = 23 per group). The α error was 0.05, and the power was 0.8. Numerical and categorical patient characteristics were compared between the groups using ANOVA and chi-square test. A linear mixed-effects model was used to analyze the hemodynamic parameters, and a post hoc analysis using the Bonferroni *t*-test was conducted. Statistical analyses were performed using SPSS Statistics version 25.0 (SPSS Inc., Chicago, IL, USA) and R software version 4.0.2 (The R Foundation, Boston, MA, USA). Statistical significance was set at *p* < 0.05.

## 3. Results

No significant differences were observed among the groups regarding anesthesia time, surgery time, eye-opening time, extubation time, total remifentanil administration, or incidence of emergence cough (Table 2). The time of LoC was longer in the R6 group than in the R12 and P groups (56.7 ± 15.5 s, 90.6 ± 13.7 s, 66.7 ± 18.5 s, respectively, *p* < 0.001). Injection pain was absent in the R6 and R12 groups; however, eight patients (36%) in the P group experienced it.

**Figure 2:**
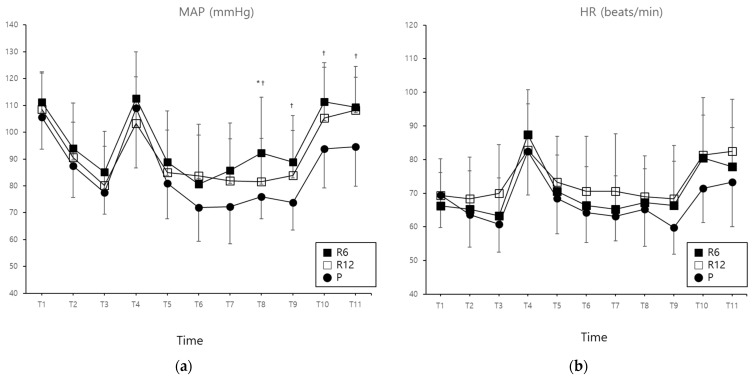
(**a**) MAP and (**b**) HR values during anesthesia; T1: baseline; T2: 1 min after anesthetic induction; T3: immediately before intubation; T4: 1 min after intubation; T5-T8: 5 min intervals during anesthesia; T9: end of operation; T10: 1 min after extubation; T11: end of anesthesia. * *p* < 0.05 vs. R12 group; ^†^ *p* < 0.05 vs. P group. MAP = mean arterial pressure; HR = heart rate.

ΔMAP between the P, R6, and R12 groups (43.8 ± 13.8 mmHg, 39.2 ± 14.3 mmHg, and 39.2 ± 13.5 mmHg, respectively, *p* = 0.443) was comparable. The change in MAP over time among the groups was not significantly different (*p* = 0.0547). However, MAP was significantly higher in the R6 group than in the R12 and P groups at T8 and in the R6 and R12 groups than in the P group from T9 to T11. The change in heart rate over time was significantly different among the groups (*p* = 0.0205) (Figure 2).

In the P, R6, and R12 groups, the frequencies of intraoperative vasoactive drug use were 54.5%, 17.4%, and 30.4%, respectively (*p* = 0.029). The median (interquartile range) dose of ephedrine used among the P, R6, and R12 groups (3 (0–6) mg, 0 (0–0) mg, and 0 (0–0) mg, respectively) was significantly different (*p* = 0.034) and significantly higher in the P group than in the R6 group (Table 3). 

## 4. Discussion

The findings of this study indicated that the R6 group had a significantly lower utilization frequency of vasoactive drugs and a lower dose of ephedrine than the propofol group, although the decrease in MAP (ΔMAP) was comparable during propofol and remimazolam anesthesia with an induction dose of 6 or 12 mg/kg/h in older patients. Moreover, the recovery profiles were comparable after propofol and remimazolam anesthesia in older patients.

Previous studies have reported inconsistent results regarding the hemodynamic changes between propofol and remimazolam anesthesia and sedation in older patients [12,13,16]. Several studies have shown that remimazolam demonstrates superior hemodynamic stability compared to propofol in older patients. The report by Zhang et al. showed that remimazolam induction of 0.2–0.4 mg/kg led to a lesser MAP decrease compared to propofol induction of 1.5–2.0 mg/kg during hip replacement [12]. Guo et al. observed that the frequency of intraoperative decrease in MAP was lower with a remimazolam dose of 0.15 mg/kg compared to a propofol dose of 1.5 mg/kg during sedation for gastrointestinal endoscopy [13]. In these studies, propofol was mainly administered as a bolus rather than an infusion. However, Sekiguchi et al. [16] reported that by using 3 µg/mL of propofol TCI as an induction dose, the induction of anesthesia with remimazolam and propofol TCI did not result in any significant hemodynamic differences. They pointed out that hemodynamics may vary depending on how the drug is injected [16]. These results are partially consistent with those of our study. In our study, when comparing propofol induction of 4 µg/mL using TCI to remimazolam induction of 6 or 12 mg/kg/h, three groups showed comparable ΔMAP during the surgery in older patients. 

In our study, the frequency of using vasoactive drugs was statistically significantly different between the R6 and propofol groups, which means that there was a difference in the frequency of hypotension and bradycardia in patients who met the initially set hypotension and bradycardia criteria. These results are comparable to those reported by Lu et al. [18]. According to their report, the remimazolam group (300 mg/h) had a lower incidence of hypotension and usage of vasoactive drugs compared to the propofol group (3 g/h) during deep sedation for gastrointestinal endoscopy when administered sedatives until the Modified Observer’s Assessment of Alertness/Sedation Scale reached ≤1. 

According to previous studies, organ injury can occur if the MAP is maintained at less than 80 mmHg for >10 min during surgery. The risk increases if the time increases or MAP decreases to less than 55 mmHg [2]. Intraoperative hypotension during non-cardiac surgery may increase the risk of major adverse cerebrovascular or cardiac events within 30 days [19]. Because these risks can be fatal for older patients, thorough monitoring and management were performed to prevent hypotension during surgery, and active intervention was carried out to prevent the patient’s hypotension from persisting during surgery. This might be one of the possible explanations for the lack of difference in ΔMAP between remimazolam and propofol anesthesia in our study. 

Previous studies that compared the effects of remimazolam and propofol anesthesia on emergence and recovery profiles showed variable results. In a study of older patients undergoing hip replacement, no significant differences were observed in the time to emergence or extubation between the groups administered remimazolam and propofol anesthesia [12]. These results are consistent with those of this study. In contrast, Doi et al. [9] reported that the mean time to eye-opening and extubation was longer after remimazolam anesthesia than after propofol anesthesia (mean age 56 years). They suggested that the recovery time of remimazolam should be considered with some caution owing to limited experience in tapering the drug. Choi et al. [20] found that patients were more heavily sedated upon admission to the postoperative care unit after remimazolam anesthesia than after propofol anesthesia (20–65 years). However, the total quality of the recovery-15 score under remimazolam anesthesia was comparable to that under propofol anesthesia. 

The offset of drug effects may be influenced by several factors, including clearance, volume of distribution, and terminal half-life. Remimazolam has a low steady-state volume of distribution (35 L), which is 1/10 times that of propofol [21]. A smaller steady-state volume of distribution is associated with faster drug elimination and patient recovery. Propofol has a slightly higher estimated total body clearance than remimazolam; however, unlike propofol, remimazolam undergoes nonspecific esterase-mediated metabolism independent of the organs. As the clearance of remimazolam is not affected by liver or kidney dysfunction [22], it may be a suitable drug for older patients with possible hepatic or renal dysfunction. The simulated context-sensitive decrement times for remimazolam were comparable to those for propofol. The decrement time of plasma concentration for remimazolam is shorter than that for propofol; however, the decrement time of the effective concentration for remimazolam is approximately 3–4 min longer than that for propofol [7]. Therefore, further studies are needed to elucidate the clinical recovery profiles after remimazolam anesthesia compared with propofol anesthesia in the older population.

This study had some limitations. First, for safety reasons, older patients with ASA 3 or higher physical status were excluded from the study. As older patients with ASA 1 or 2 physical status do not represent the older population, various patients should be included to evaluate the efficacy of remimazolam. Second, the combination of propofol and remifentanil is known to have a synergistic effect [23,24]; however, data on the interaction between remimazolam and remifentanil are insufficient. Therefore, further studies are required to elucidate this mechanism. Finally, when flumazenil is used for remimazolam reversal, the recovery parameters would be different because remimazolam–flumazenil anesthesia would provide faster recovery than propofol anesthesia [25].

In conclusion, this study showed that remimazolam anesthesia with an induction dose of 6 mg/kg/h, rather than 12 mg/kg/h, could reduce the requirement for intraoperative vasoactive drugs and ephedrine usage compared to propofol anesthesia using TCI. However, ΔMAP and recovery profiles were comparable between propofol and remimazolam anesthesia in older patients.

## Figures and Tables

**Table 1:** Patients’ characteristics.

	P Group (*n* = 22)	R6 Group (*n* = 23)	R12 Group (*n* = 23)
Age, years	68 (65–82)	73 (65–86)	72 (65–81)
Gender (M/F)	11/11	12/11	17/6
Height, cm	160.9 ± 8.8	160.0 ±9.0	160.7 ± 8.2
Weight, kg	62.0 ± 12.0	61.0 ± 12.8	61.5 ± 9.9
Body mass index, kg/m^2^	23.9 ± 3.3	23.8 ± 4.2	23.7 ± 2.8
ASA physical status, I/II	4/18	6/17	9/14
Type of surgery			
Nasal	8 (36)	5 (23)	7 (32)
Throat	5 (32)	8 (36)	6 (27)
Urology	7 (23)	7 (32)	8 (36)
Hepatobiliary	1 (5)	2 (9)	1 (5)
Breast	1 (5)	1 (5)	1 (5)

Values are presented as mean ± standard deviation, median (range), or number (%).

**Table 2:** Anesthetic profile.

	P Group (*n* = 22)	R6 Group (*n* = 23)	R12 Group (*n* = 23)	*p*-Value
LoC time, s	56.7 ± 15.5 ^†^	90.6 ± 13.7 *^,‡^	66.7 ± 18.5 ^†^	<0.001
LoC dose, mg/kg	90.9 ± 16.0	12.2 ± 10.6	15.2 ± 5.1	NA
Injection pain	8 (36)	0 (0) *	0 (0) *	<0.001
Total infused dose, mg/kg	7.9 ± 2.6	0.9 ± 0.37	1.1 ± 0.4	<0.001
Total remifentanil dose, µg/kg	7.6 ± 3.3	5.9 ± 4.0	6.8 ± 3.3	0.265
Surgery time, min	42.0 ± 23.6	32.8 ± 18.8	35.7 ± 21.6	0.342
Anesthesia time, min	77.0 ± 24.9	63.9 ± 19.0	77.2 ± 31.5	0.139
Eye opening time, min	10.0 ±3.1	13.0 ± 6.1	12.3 ± 4.2	0.095
Extubation time, min	11.0 ± 3.1	13.9 ± 5.9	13.2 ± 4.3	0.102
Emergence cough, 0/1/2/3	6/12/3/1	8/11/2/2	7/8/2/6	0.684
Fluid, mL	304.5 ± 158.8	280.4 ± 138.8	279.5 ± 199.2	0.853
Blood loss, mL	187.5 ± 170.6	113.8 ± 85.8	112.9 ± 131.9	0.464

Values are presented as mean ± standard deviation or number (%). Emergence cough; 0 = no cough, 1 = single cough, 2 = more than one episode of non-sustained cough, 3 = sustained and repetitive cough with head lift. * *p* < 0.05 vs. P group; ^†^ *p* < 0.05 vs. R6 group; ^‡^ *p* < 0.05 vs. R12 group. NA = not applicable.

**Table 3:** Decrease in mean arterial pressure and number of vasoactive drugs during anesthesia.

	P Group (*n* = 22)	R6 Group (*n* = 23)	R12 Group (*n* = 23)	*p*-Value
MAP decrease, mmHg	43.8 ± 13.8	39.2 ± 14.3	39.2 ± 13.5	0.443
Vasoactive drugs, *n*	12	4 *	7	0.029 *
Ephedrine, *n*	11	4	5	
Phenylephrine, *n*	0	0	0	
Glycopyrrolate, *n*	1	1	2	
Atropine, *n*	0	0	1	
Dose of ephedrine, mg	3 (0–6)	0 (0–0) *	0 (0–0)	0.034

Values are presented as mean ± standard deviation, median [interquartile range], or number. * *p* < 0.05 vs. P group.

## Data Availability

The study’s data are available on request from the corresponding author.
